# Hysteria in the Male

**Published:** 1889-12-21

**Authors:** 


### HYSTERIA IN THE MALE.

Two cases of hysteria in the male occurring at the Paddington Infirmary recently find a notice in the *Lancet.* A valet, aged 30, was admitted in July last, complaining of general weakness, occasional sickness, giddiness, and neuralgic pains in various parts. The thoracic and other viscera and organs were normal, but there was hyperœthesia of the abdomen, especially about what is known in the female as the ovarian region. Pressure in this part gave rise to a choking sensation in the throat. On the field of vision being tested there was found a well-marked general retraction. He had been long subject to nervous attacks. An attack commenced with profuse perspiration, then he would fall down, though without complete loss of consciousness, and after about ten minutes he came round, crying or laughing. On one or two occasions he passed involuntarily, and often ground his teeth, but did not bite his tongue. There was a previous history of excess, and a family history of the hysterical diathesis.

The second case presented in a man aged 55, admitted in June last. He appeared at first sight very ill, and wore a most distressed look in keeping with the gloomy view he took of life. He said his right leg had been paralysed owing to an injury received twenty-four years ago, causing him to walk with a stick, and that the left leg had become affected without cause, although he had carried on his business as a greengrocer. All his life he had been very nervous, and lately been subject to hot flushings, followed by cold shivers. It was found he had flaccid paresis of both lower extremities, but not complete in either. Attacks would come on after some excitement. The face and sometimes other parts of the body became red and looked hot, although not feeling so, then there would be profuse general perspiration. The viscera were healthy, and previous and family history were negative. There was no inguinal tenderness. In the first case no specific line of treatment was adopted, but he left the infirmary well. In the second case limits were placed on the visits of sympathising friends, and the patient was persuaded he could walk if he tried. Liberal diet, bark, and acid were given. After two months' treatment he was nearly well. It may, however, be, perhaps, doubted if there will not be a relapse. Now, there is no particular reason why hysteria should not occur in the male, for the idea that it is always related to uterine and ovarian disturbance in the female is not correct. As Dr. Buzzard states, this connection is neither more nor less than that which obtains in other neuroses.

